# Increased role of E prostanoid receptor-3 in prostacyclin-evoked contractile activity of spontaneously hypertensive rat mesenteric resistance arteries

**DOI:** 10.1038/s41598-017-09288-w

**Published:** 2017-08-21

**Authors:** Bin Liu, Mengyi Zhan, Yingzhan Zhang, Hui Li, Xiangzhong Wu, Fengfeng Zhuang, Wenhong Luo, Yingbi Zhou

**Affiliations:** 1 Cardiovascular Research Center, Shantou University Medical College, Shantou, China; 2 The Central Lab, Shantou University Medical College, Shantou, China; 3 Beijing View Solid Biotechnology, Beijing, China

## Abstract

This study aimed to determine whether E prostanoid receptor-3 (EP3) is involved in prostacyclin (PGI_2_)-evoked vasoconstrictor activity of resistance arteries and if so, how it changes under hypertensive conditions. Mesenteric resistance arteries from Wistar-Kyoto rats (WKYs) and spontaneously hypertensive rats (SHRs) were isolated for functional and biochemical studies. Here we show that in vessels from WKYs, PGI_2_ or the endothelial muscarinic agonist ACh (which stimulates *in vitro* PGI_2_ synthesis) evoked vasoconstrictor activity, which increased in SHRs. The thromboxane-prostanoid receptor (TP) antagonist SQ29548 partially removed the vasoconstrictor activity, and an increased contractile activity of PGI_2_ resistant to SQ29548 was observed in SHRs. Interestingly, L798106, an antagonist of EP3 (whose expression was higher in SHRs than in WKYs), not only added to the effect of SQ29548 but also caused relaxation to PGI_2_ more than that obtained with SQ29548. In accordance, EP3 deletion, which reduced PGI_2_–evoked contraction, together with SQ29548 resulted in relaxation evoked by the agonist in mouse aortas. These results thus demonstrate an explicit involvement of EP3 in PGI_2_-evoked vasoconstrictor activity in rat mesenteric resistance arteries and suggest that up-regulation of the receptor contributes significantly to the increased contractile activity evoked by PGI_2_ under hypertensive conditions.

## Introduction

Metabolism of arachidonic acid via cyclooxygenase (COX), which includes COX-1 and -2, produces vasoactive prostanoids. Among them, thromboxane A_2_ (TxA_2_) is the major product produced in platelets, and it acts on the Tx prostanoid receptor (TP) to mediate vasoconstrictor and platelet-aggregating effects. In contrast, prostacyclin (prostaglandin I_2_; PGI_2_) is mainly produced in vascular endothelium and is proposed to act on the I prostanoid receptor (IP) that mediates vasodilatation and opposes the effects of TP^1–5^. However, in some vascular beds, including those of humans, PGI_2_ or endothelial COX-derived metabolites evoke vasoconstrictor response^6–20^. This has been explained by a concurrent modulation of PGI_2_’s vasomotor reaction via IP and TP; an endothelium-derived contracting factor (EDCF)-like action of PGI_2_ or a vasoconstrictor response evoked by endothelial COX metabolites (which consist mainly of PGI_2_) can reflect limited expression or function of IP, which leads to uncovering of the vasoconstrictor activity derived from concurrently activated TP^21–26^.

More importantly, endothelial COX-derived vasoconstrictor activity or EDCF-like action of PGI_2_ plays an important role in the development of endothelial dysfunction under disease conditions, including hypertension^27–33^. It has been suggested that in hypertension or under prehypertensive conditions, endothelial PGI_2_ synthesis may increase^25, 34^. Also, under the disease condition, IP is suggested to become dysfunctional^24, 25^. These findings explain why an increased vasoconstrictor response and/or a conversion of dilator responses evoked by endothelial PGI_2_ synthesis into contractile responses were observed in prehypertensive or hypertensive conditions^24, 25, 27, 34^. As a result, TP antagonism could be used as an effective remedy for endothelial dysfunction developed under disease conditions^24, 27^. At the same time, our recent studies suggest that PGI_2_ also activates the E prostanoid receptor-3 (EP3; a vasoconstrictor receptor of PGE_2_), which along with TP accounts for the EDCF-like action of PGI_2_^35^. Interestingly, the EDCF activity or PGI_2_-evoked contractile activity in Wistar-Kyoto (WKY) or spontaneously hypertensive rat (SHR) vessels has been recognized to contain a component of TP-independent mechanism^18, 29, 34^, which favors an involvement of EP3 that should also be targeted under hypertensive conditions. Indeed, EP3 knockout (EP3^−/−^) has been found to attenuate ANG II pressor response^36^. However, the role of EP3 in the EDCF-like action of PGI_2_ in resistance arteries and how it changes under hypertensive conditions still remain to be elucidated.

Therefore, in this study WKY and SHR mesenteric resistance arteries were isolated for biochemical and/or functional analyses. In addition, a strain of EP3^−/−^ mice was designed and used to explicitly elucidate the role of the receptor in the vasoconstrictor activity of PGI_2_ under *in vitro* conditions.

## Results

### Response evoked by PGI_2_ and effect of TP and/or EP3 antagonism

**Figure 1:**
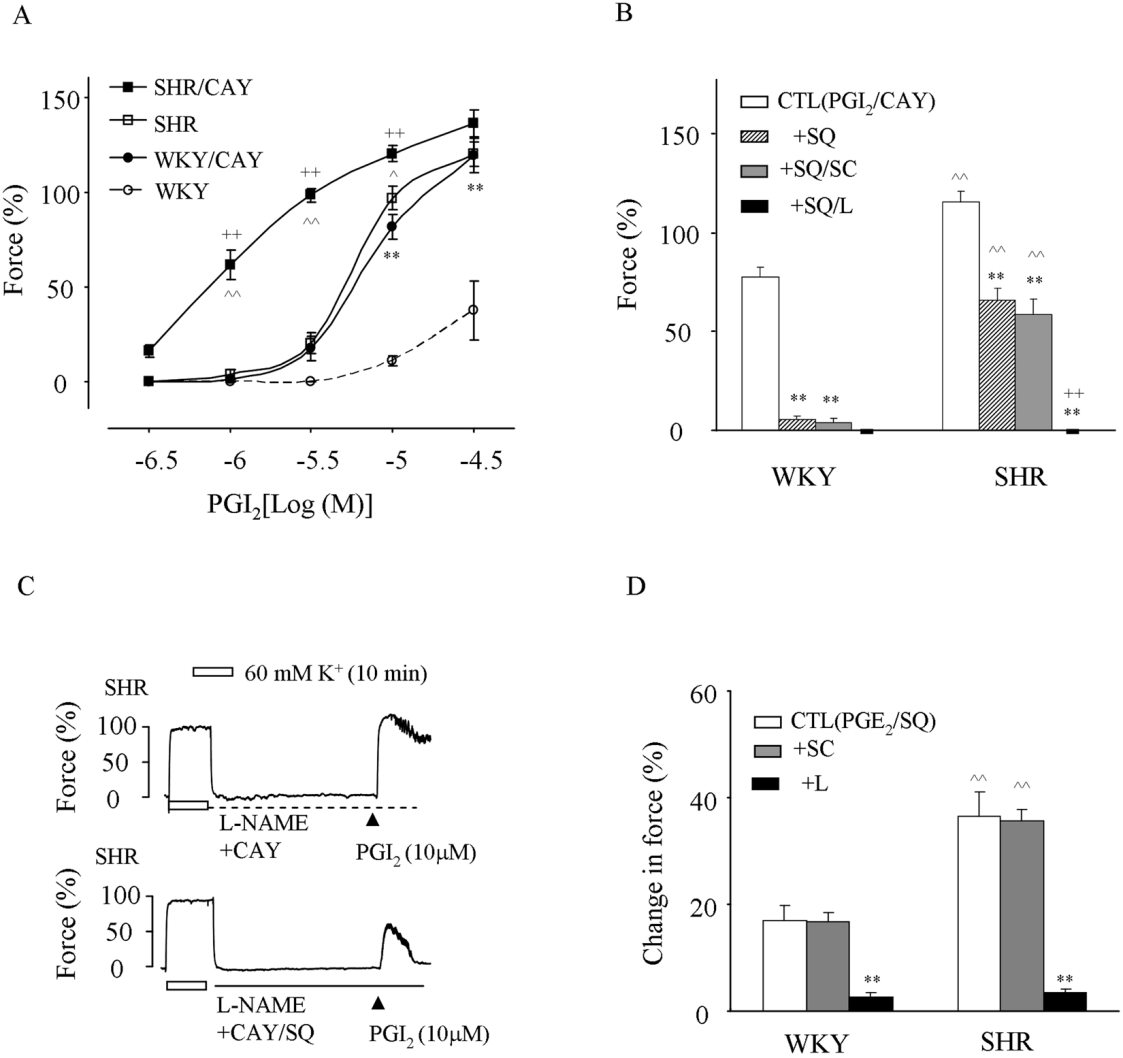
PGI_2_-evoked contraction in NOS-inhibited mesenteric resistance arteries and effect of EP3 antagonism. (**A**) Comparison of responses evoked by PGI_2_ in L-NAME-treated WKY and SHR vessels with or without the presence of the IP antagonist CAY10441 (1 μM; CAY). N = 5 for each; ^**^ *P* < 0.01 vs. WKY; ^^^ *P* < 0.05 and ^^^^ *P* < 0.01 vs. SHR; ^++^ *P* < 0.01 vs. WKY/CAY. (**B**) Comparison of the control contraction evoked by PGI_2_ (CTL; 10 μM) in WKY or SHR vessels obtained with CAY with that additionally with the TP antagonist SQ29548 (10 μM; +SQ), that with SQ29548 and the EP1 antagonist SC19220 (10 μM; +SQ/SC), or that with SQ29548 and the EP3 antagonist L798106 (1 μM; +SQ/L). (**C**) Representative traces showing the control contraction evoked by PGI_2_ (10 μM) in SHR vessels obtained with CAY (top) and that the additionally with the TP antagonist SQ29548 (10 μM; bottom). (**D**) Summary of control responses evoked by PGE_2_ (0.1 μM) on the contraction evoked by PE (3 μM) in L-NAME and SQ29548-treated WKY and SHR vessels and those obtained additionally with SC19220 (+SC) or L798106 (1 μM;+SQ/L). In (**B**) and (**D)**, n = 5 for each; ^**^ *P* < 0.01 vs. CTL; ^^^^ *P* < 0.01 vs. WKY counterparts; ^++^ *P* < 0.01 vs. +SQ.

The responses to PGI_2_ were first examined in mesenteric resistance arteries treated with the NO synthase (NOS) inhibitor N^ω^-nitro-L-arginine methyl ester (L-NAME) under baseline conditions^12, 25^. As shown in Fig. 1A, in WKY vessels, PGI_2_ caused a slight contractile response only at concentrations ≥10 μM. However, in those of SHRs, not only the initial concentration of PGI_2_ to evoke contractile response was lower, but also the extent of contraction evoked by the agonist was significantly increased compared to that in WKYs (Fig. 1A). The IP antagonist CAY10441 (1 μM) significantly increased the contraction evoked by PGI_2_ both in WKYs and in SHRs (Fig. 1A). In addition, under such conditions, the response evoked by PGI_2_ in SHRs was still greater than that in WKYs (Fig. 1A).

Moreover, we noted that after the treatment with CAY10441, the TP antagonist SQ29548 (10 μM), abolished most of the contraction evoked by 10 μM PGI_2_ in WKYs, but <50% of that in SHRs (Fig. 1B and C). The EP3 antagonist L798106 (1 μM) but not the EP1 antagonist SC19220 (10 μM) added to the effect of SQ29548, resulted in abolition of the contraction evoked by PGI_2_ in both WKYs and SHRs (Fig. 1B). In addition, under NOS-inhibited conditions, PGE_2_ (which evokes only a minor vasoconstrictor activity that peaks at concentrations ≤0.3 μM in TP^−/−^ mouse aortas^35^) was able to evoke an increase of force at 0.1 μM on the contraction evoked by 3 μM phenylephrine (PE) when SQ29548 was also present (Fig. 1D). Again, this increase of force was more prominent in SHRs than in WKYs and was inhibited by L798106 (1 μM) but not by SC19220 (10 μM; Fig. 1D).

### Effect of TP and/or EP3 antagonism on PGI_2_-response under precontracted conditions

**Figure 2:**
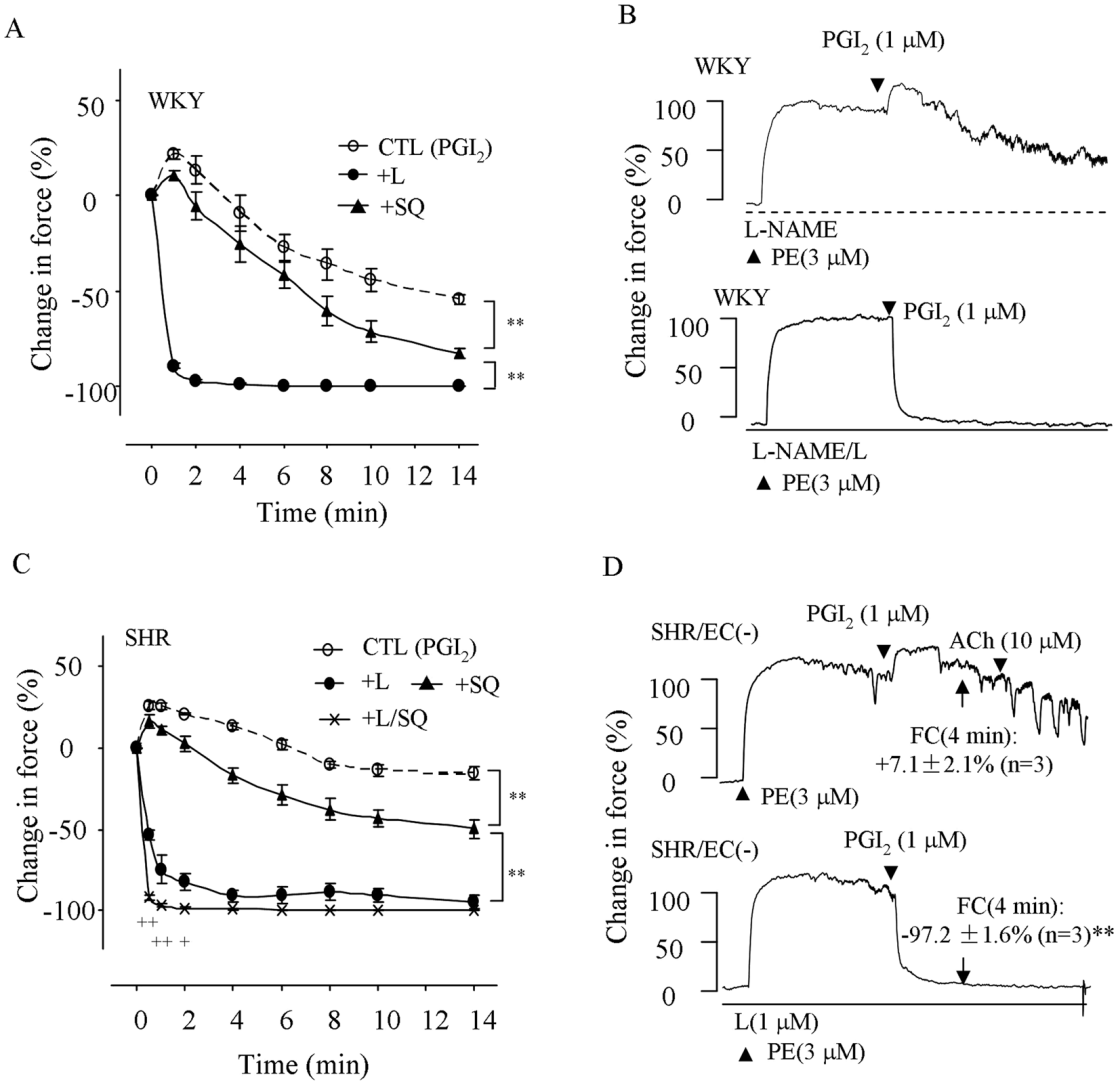
Effect of TP and/or EP3 antagonism in PE-precontracted, NOS-inhibited or endothelium-denuded rat mesenteric resistance arteries. (**A**) The control response evoked by PGI_2_ (1 μM; CTL) in L-NAME-treated WKY vessels precontracted with PE (3 μM) and that obtained with the EP3 antagonist L798106 (1 μM; +L; bottom) or the TP antagonist SQ29548 (+SQ; 10 μM). (**B**) Representative traces showing the response evoked by PGI_2_ (top) and that obtained with L798106 (1 μM; +L; bottom) in (**A**). (**C**) The control response evoked by PGI_2_ in PE-precontracted, L-NAME-treated SHR vessels and that obtained with SQ29548 (+SQ), with L798106 (+L), or with L798106 and SQ29548 (+L/SQ). In A and C, n = 5 for each; ^**^ *P* < 0.01; ^+^ *P* < 0.05 and ^++^ *P* < 0.01 vs. the value in +L. (**D**) Representative traces with summarized values showing the control response evoked by PGI_2_ (top) in PE-precontracted, endothelium-denuded SHR vessels (top) and that obtained with L798106 (+L; bottom). FC (4 min): the value of force change relative to that of PE 4 min after the application of PGI_2_. ^**^ *P* < 0.01.

The effect of TP or EP3 antagonism on the response evoked by PGI2 was also determined in L-NAME-treated mesenteric resistance arteries precontracted with PE (3 μM). As shown in Fig. 2, in vessels of either rat strain, PGI2 (1 μM) evoked an initial increase of force, which was followed by a steady relaxation that was to a smaller extent in SHRs than in WKYs (−16.0 ± 4.3% vs. −54.8 ± 6.4%, respectively; P < 0.01).

As expected, the TP antagonist SQ29548 (10 μM) enhanced the relaxation (Fig. 2A and C), with resulting force being smaller in WKYs than in SHRs (−83.3 ± 3.7% vs. −49.9 ± 5.6%, respectively; P < 0.01). Interestingly, the EP3 antagonist L798106 (1 μM) abolished the initial increases of force and yielded greater relaxations than that obtained with SQ29548 (Fig. 2A,B and C) In addition, in SHRs where L798106 (1 μM) did not cause a complete relaxation, SQ29548 (10 μM) added to the effect of L798106 and resulted in a complete relaxation evoked by PGI_2_ (Fig. 2C).

The effect of L798106 on PGI_2_-evoked response was also determined in endothelium-denuded, PE (3 μM)-precontracted SHR mesenteric resistance arteries. Under the conditions PGI_2_ evoked a response similar to that obtained in L-NAME-treated SHR vessels (Fig. 2D). Again, L798106 removed the initial force increase and caused an enhanced relaxation to PGI_2_, i.e., the force change (relative to the value of PE pre-contraction) 4 min after the application of PGI_2_ [FC (4 min)] being significantly smaller than that of controls (Fig. 2D).

### Expressions and/or functions of EP3, TP and IP

To understand molecular bases for above results, expressions of EP3, TP and IP were examined. Real-time PCR showed that the level of EP3 mRNAs normalized by that of β-actin was higher in SHRs than in WKYs. Also, Western blot revealed that β-actin normalized level of TP proteins was increased, while that of IP was decreased in SHRs than in WKYs.

**Figure 3:**
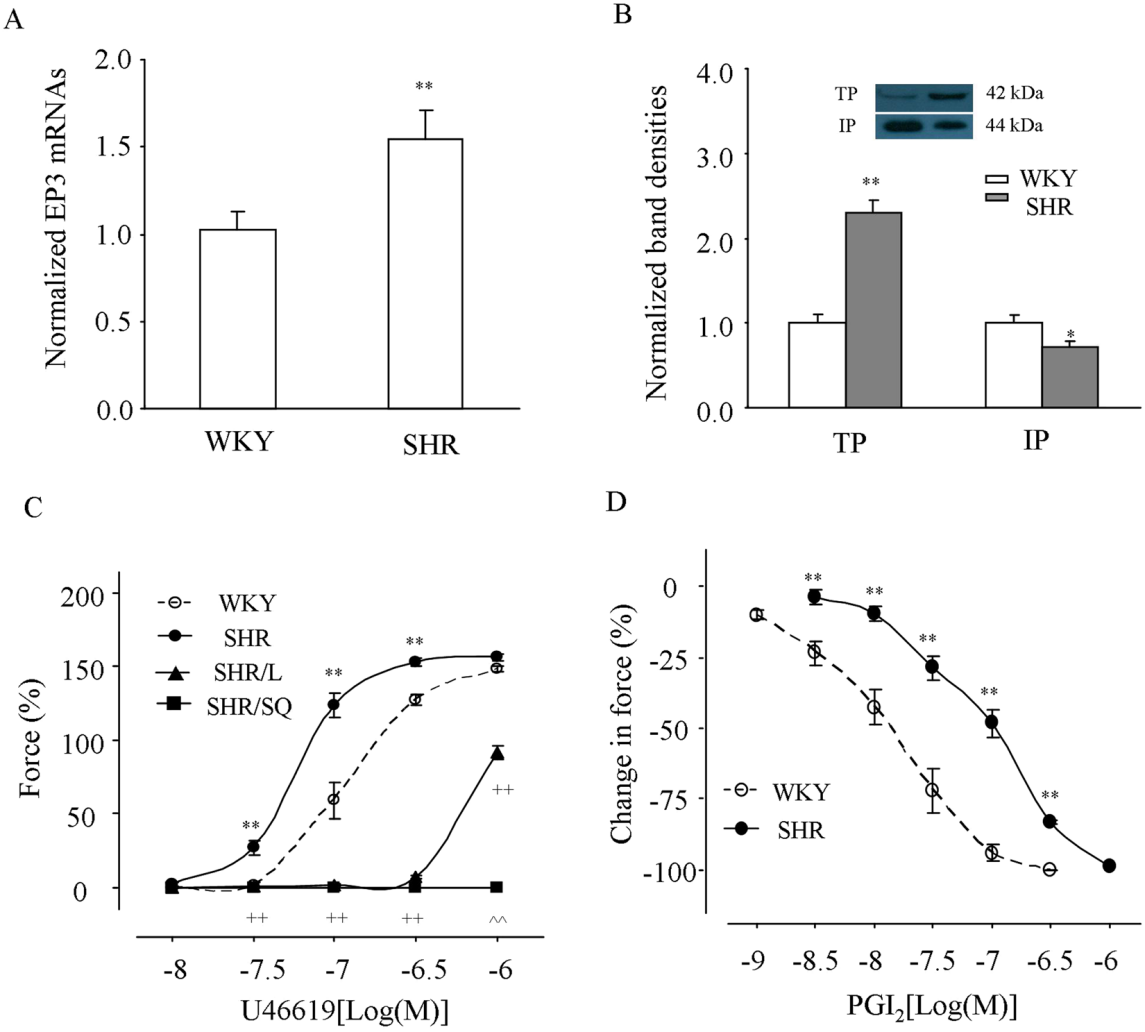
Expressions and/or functions of EP3, TP and IP. (**A**) β-actin normalized EP3 mRNA level detected by real-time PCR in WKY and SHR vessels. N = 6 for each. (**B**) Representative Western blots of TP and IP (left lane: WKY; right lane: SHR) and summary of results (from 5 replicates) showing the band density of TP or IP normalized by that β-actin in WKY and SHR mesenteric resistance arteries. Full length representative Western blots of TP and IP bands and that of β-actin were presented in the supplementary file. In (**A**) and (**B**), the normalized EP3 mRNA level, TP and IP band densities were expressed relative to the average of WKY vessels (which was assumed a value of 1.0). ^*^ *P* < 0.05 and ^**^ *P* < 0.01 vs. the value in WKY. (**C**) Contractions evoked by the TP agonist U46619 in L-NAME-treated WKY and SHR vessels and that of SHR vessels obtained with TP antagonist SQ29548 (10 μM; SHR/SQ) or the EP3 antagonist L798106 (1 μM; SHR/L). (**D**) PGI_2_-evoked relaxation in L-NAME-treated WKY and SHR vessels precontracted with PE (3 μM) after both EP3 and TP were antagonized (with L798106 and SQ29548). In (**C)** and (**D**), n = 5 for each; ^**^ *P* < 0.01 vs. WKY; ^++^ *P* < 0.01 vs. SHR; ^^^^P < 0.01 vs. SHR/L.

Also, we noted that the TP agonist U46619 was found to evoke a greater contraction in SHR than in WKY vessels (Fig. 3C). The TP antagonist SQ29548 (10 μM) completely abolished the contraction in SHRs. Notably, although the EP3 antagonist L798106 (1 μM) also inhibited the response, its effect was to a smaller extent than that of SQ29548 (Fig. 3C). In addition, after vessels had been treated with L798106 and SQ29548, the relaxation evoked by PGI_2_ on contractions induced by PE (3 μM) was smaller in SHRs than that in WKYs (Fig. 3D).

### ACh-evoked responses in mesenteric resistance arteries

**Figure 4:**
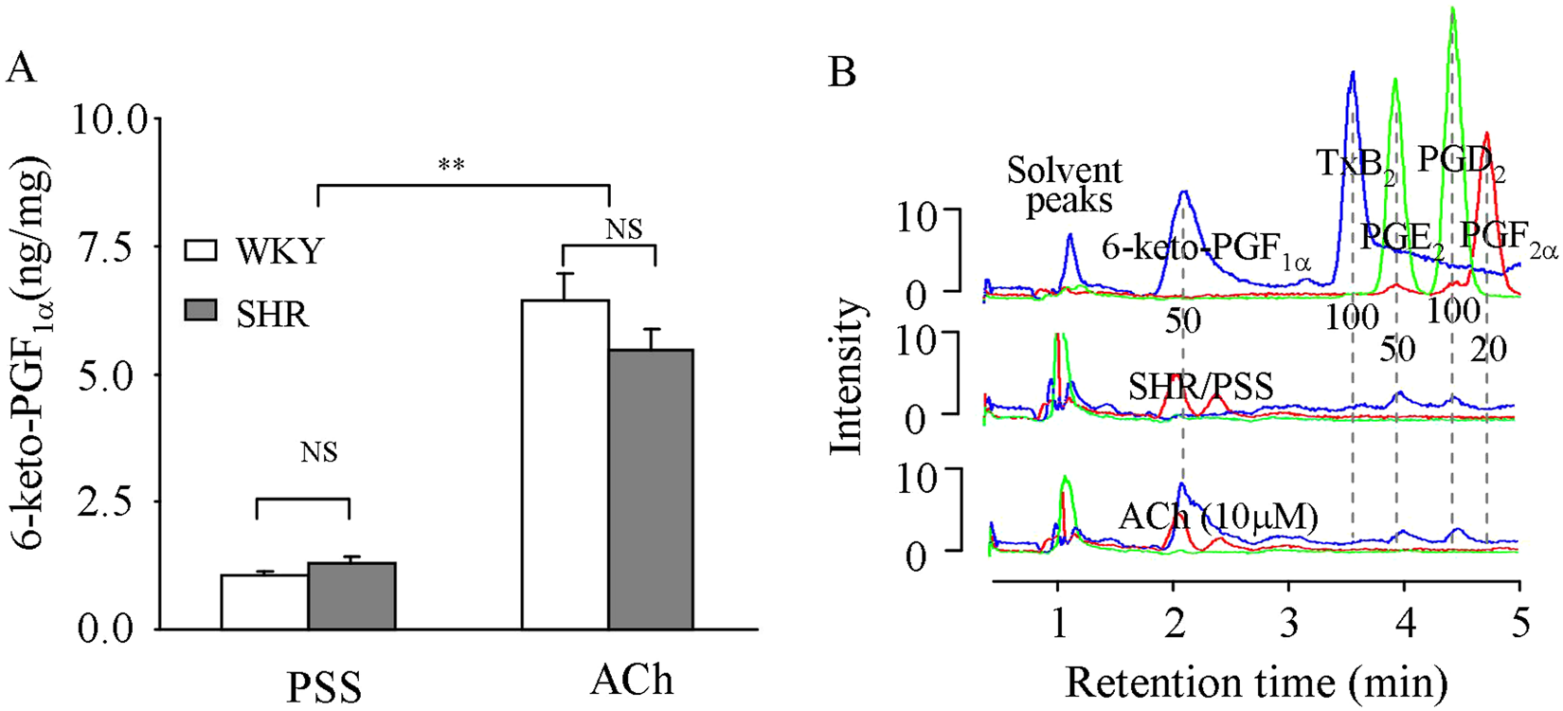
ACh-evoked production of 6-keto-PGF_1α_ in rat mesenteric resistance arteries. (**A**) production of 6-keto-PGF_1α_ measured with EIA in WKY and SHR vessels under the basal (un-stimulated in PSS) and ACh-stimulated conditions. N = 7 for each; ^**^ *P* < 0.01; NS: not significant. (**B**) HPLC-MS showing peaks of 6-keto-PGF_1α_ and other products in basal (middle) or ACh-stimulated SHR vessels (bottom). The top shows peaks obtained with solution of mixed standard compounds with amount indicated (ng/ml).

The muscarinic agonist ACh stimulates endothelial COX to produce PGI_2_^12, 34^. Therefore, the production of native PGI_2_ in WKY and SHR mesenteric resistance arteries was examined. As shown in Fig. 4A, in WKY and SHR vessels, ACh (10 μM) stimulated an increase in the production of the PGI_2_ metabolite 6-keto-PGF_1α_ that was comparable between the two rat strains. HPLC-MS further revealed that 6-keto-PGF_1α_ was the only detected COX-derived product in ACh-stimulated SHR vessels (Fig. 4B).

**Figure 5:**
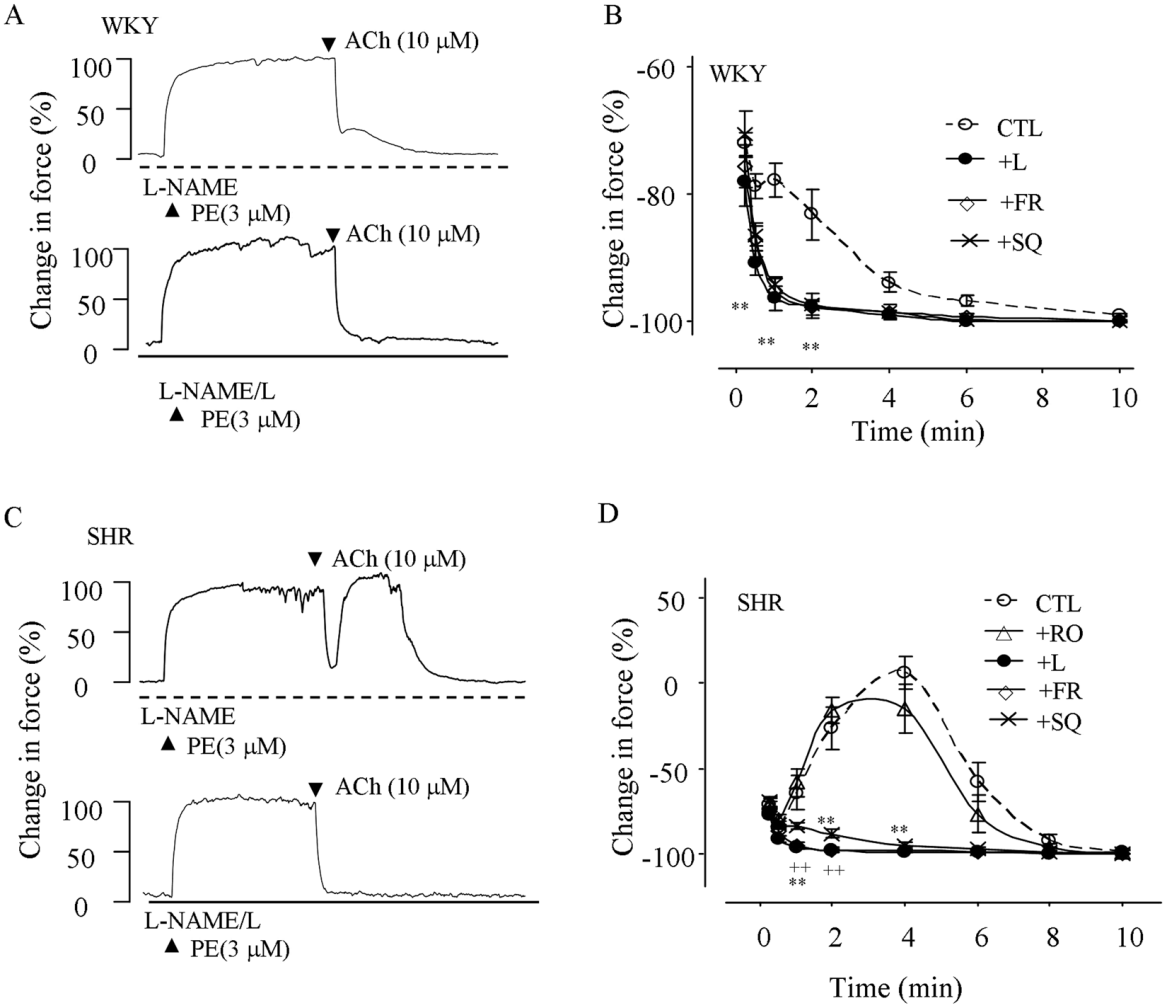
ACh-evoked responses in NOS-inhibited rat mesenteric resistance arteries precontracted with PE. (**A**) Representative traces showing the control response (CTL) evoked by ACh (10 μM; top) in L-NAME-treated WKY vessels precontracted with PE (3 μM) and that obtained with the EP antagonist L798106 (1 μM; +L; bottom). (**B**) Summary of results from 5 replicates in (**A)**, and those obtained with the TP antagonist SQ29548 (10 μM; +SQ) or the COX-1 inhibitor FR122047 (1 μM; +FR). (**C**) Representative traces showing the control response (CTL) evoked by ACh (top) in PE-precontracted, L-NAME-treated SHR vessels and that obtained with L798106 (+L; bottom). (**D**) Summary of results from 5 replicates in (**C**) and those obtained with SQ29548 (+SQ), FR122047 (+FR), or the COX-2 inhibitor rofecoxib (1 μM; +RO). In (**B**) and (**D**), ^**^ *P* < 0.01 vs. CTL while ^++^ *P* < 0.01 vs. +SQ.

Vasomotor reactions evoked by ACh (10 μM) were also determined. Vessels were again treated with L-NAME^12, 25, 34^. As shown in Fig. 5, when vessels were precontracted with PE (3 μM), ACh (which was unable to evoke any response under baseline conditions) evoked relaxation, which was blunted by a biphasic force that was more prominent in SHRs than in WKYs (peak of the force was 5.6 ± 9.7% above PE-evoked response vs. −77.9 ± 2.6%, respectively; P < 0.01; Fig. 5). The COX-1 inhibitor FR122047 (1 μM) and the EP3 antagonist L798106 (1 μM) similarly abolished the biphasic force (Fig. 5). In contrast, the TP antagonists SQ29548 (10 μM), although it abolished the biphasic force in WKYs (Fig. 5B), showed an effect in SHRs less than that of L798106 (Fig. 5D). Also, the COX-2 inhibitor rofecoxib (1 μM) did not show an effect in SHR vessels (Fig. 5D).

### Effect of EP3^−/−^ on contractile response to PGI_2_

**Figure 6:**
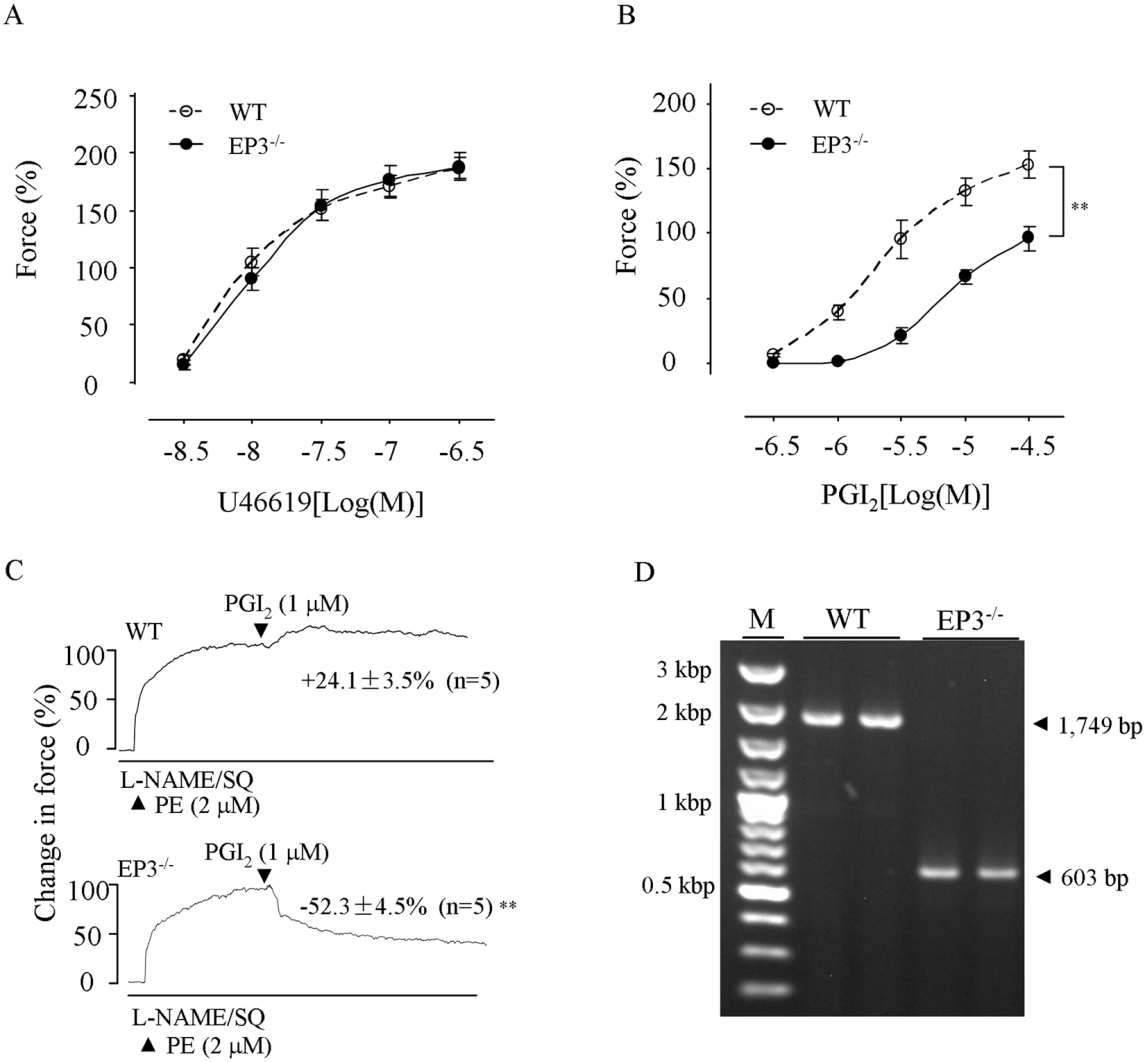
Effect of EP3^−/−^ on responses evoked by PGI_2_ in NOS-inhibited mouse abdominal aortas. (**A**) and (**B**) Responses evoked by the TP agonist U46619 (**A**) and PGI_2_ (**B**) in WT and EP3^−/−^ abdominal aortas. (**A**) representative traces with summarized values showing the response evoked by PGI_2_ in WT (top) or EP3^−/−^ abdominal aortas (bottom) with the presence of TP antagonist SQ29548 (10 μM; SQ). In **A**–**C**, n = 5 for each; ^**^ *P* < 0.01 vs. WT. (**D)** Genotyping showing the difference in the PCR product of EP3 gene between WT and EP3^−/−^ mice. M: size marker.

Finally, abdominal aortas (which normally show contraction to PGI_2_^12, 35^) from EP3^−/−^ mice were used to verify the role of EP3 in PGI_2_-evoked contractile activity. As shown in Fig. 6A and B, in L-NAME-treated abdominal aortas of EP3^−/−^ mice, the response evoked by U46619 was unaltered (Fig. 6A); however, that of PGI_2_ was reduced compared to WT mice (Fig. 6B). Moreover, in such vessels pre-contracted with PE (2 μM), treatment with the TP antagonist SQ29548 (10 μM) resulted in relaxation evoked by PGI_2_, which was in contrast to an enhancement of contraction evoked by the agonist in WT controls (Fig. 6C). Also, genotyping showed that in EP3 locus of EP3^−/−^ mice, a DNA fragment of 1146 bp was deleted as expected (Fig. 6D).

## Discussion

In this study, we show that in WKY mesenteric resistance arteries PGI_2_ evokes a vasoconstrictor activity that increases in SHRs. The TP antagonist SQ29548, which completely abolishes the response evoked by U46619 (a TxA_2_ analogue and TP agonist), only partially reduces the vasoconstrictor activity of PGI_2_. In contrast, the EP3 antagonist L798106, which also appears as a partial TP antagonist, not only adds to the effect of SQ29548, but reduces PGI_2_’s contractile activity more than SQ29548. In addition, EP3 expression level is higher in SHRs than in WKYs. These results may suggest an intimate link between EP3 and the increased vasoconstrictor activity of PGI_2_ in SHR mesenteric resistance arteries.

The responses obtained under baseline or PE-precontracted conditions clearly show that in mesenteric resistance arteries of either rat strain, PGI_2_ evokes both dilator and contractile activities, which can produce a contractile response that increases under hypertensive conditions. Interestingly, after IP (which mediates the dilator activity of PGI_2_) was blocked, 10 μM of the TP antagonist SQ29548, which has been shown to have an effect comparable to that of TP^−/−^^35^, and was able to completely abolish the response to U46619 here in SHR vessels, only partially inhibited the contractile response of PGI_2_. In addition, the part of response resistant to SQ29548 was more prominent (>50%) in SHRs than in WKYs. These results together with the antagonistic effect of L798106 (EP3 antagonist) may point to an involvement of EP3 in PGI_2_-evoked contractile activity that increases under hypertensive conditions. In its consistency, a smaller relaxation was evoked by PGI_2_ in SHR vessels than in WKY counterparts after TP had been blocked. Moreover, mRNA levels and extents of TP-independent vasoconstrictor activity of PGE_2_ sensitive to L798106 suggest that the function or expression of EP3 is up-regulated in SHR mesenteric resistance arteries.

Meanwhile, in SHR vessels the expression of TP was also increased, while that of IP was reduced compared to WKY counterparts. Accordingly, U46619 (TP agonist) evoked greater contraction, while the relaxation to PGI_2_ after both TP and EP3 had been antagonized was smaller in SHRs than in WKYs. Indeed, an increased contraction to the TP agonist and/or reduced or absent relaxation to PGI_2_ in SHR vessels has been reported previously by other groups^24, 25, 30^. Thus, TP is also up-regulated, while IP is down-regulated in SHR mesenteric resistance arteries. Such findings would not contradict with the involvement of EP3 in PGI_2_’s response. This is because results from EP3^−/−^ mice not only explicitly verify the ability of PGI_2_ to activate EP3, but along with those in TP^−/−^ mice we reported previously suggest that the agonist can activate EP3 along with TP and IP^35^. In addition, the fact that L798106 partially antagonized the contraction evoked by U46619 would not undermine its concurrent effect on EP3 as well, since its effects on both TP and EP3 explain why the antagonist resulted in relaxation to PGI_2_ more than SQ29648, yet the later added to its effect. As a result, the increased contraction to PGI_2_ observed in SHR mesenteric resistance arteries could possibly result from a synergy of effects from up-regulation of both EP3 and TP together with down-regulation of IP.

Also, under NOS inhibited conditions, the relation evoked by ACh (which concurrently stimulates release of endothelium-derived hyperpolarizing factor; EDHF^37^) was blunted by a biphasic force sensitive to COX-1 inhibition but increased in SHRs. Interestingly, PGI_2_ was found to be the major COX product evoked by ACh and as reported previously, its amount was similar between the two rat strains^30^. Thus, the vasoconstrictor activity of ACh blunting EDHF activity that increases in SHR vessels mainly results from natively produced PGI_2_ via mechanisms of the above mentioned for extraneously applied PGI_2_. In support of this idea, L798106 (EP3 antagonist), which only partially antagonized TP, removed the biphasic force as COX-1 inhibition. The fact that under PE-precontracted conditions, SQ29548 (TP antagonist) was more effective on the vasoconstrictor activity of ACh than on the response evoked by PGI_2_ could be due to the concurrent EDHF activity, which might mask any smaller contractile activity derived from native PGI_2_ resulting from one of the vasoconstrictor receptors being antagonized. Also, the concurrent EDHF activity could have possibly prevented ACh from evoking contraction under baseline conditions as we previously noted in mouse renal arteries^38^.

Therefore, our above results demonstrate an explicit involvement of EP3 in PGI_2_-evoked contractile activity and suggest that up-regulation of the receptor contributes to the increased EDCF-like action of PGI_2_ under hypertensive conditions. Interestingly, the extent of contraction evoked by PGI_2_ or endothelial COX metabolites in WKY mesenteric resistance arteries is similar to that reported in human vessels^6^, and although subtle, it can be remarkably increased under hypertensive conditions. Also, compared to that of NO, the synthesis of PGI_2_ is suggested to be less vulnerable to impairment caused by vascular pathology^39^. In addition, the amount of PGI_2_ released by endothelial stimuli such as ACh, could be higher than its minimal level to evoke vasoconstrictor activity (1 ng/mg 6-keto-PGF_1α_ could be translated into 2.7 μmol/kg PGI_2_/tissue). These facts together underscore a pathogenic role of the EDCF action of PGI_2_, especially under disease conditions. Indeed, COX-1^−/−^, which abolishes endothelial PGI_2_ synthesis although along with that of TxA_2_ in platelets, not only alleviates renovascular and diabetic hypertension, but also reduces atherosclerotic lesions^39–41^. Similar results have been obtained with COX-1 or non-selective COX inhibition when started from prehypertensive stage^34, 42^. Thus, EP3 could be an important target of pharmacological intervention along with TP for the improvement of endothelial function under disease conditions.

Similar to our present findings, an increased vasoconstrictor activity caused by PGI_2_ or endothelial COX metabolites had been reported in SHR mesenteric resistance arteries previously^29, 30^. A contraction to PGI_2_ was also reported in similar vessels of normal Sprague-Dawley rats, although the response could also be inhibited by the EP1 antagonist SC19220 (which also functions as a partial TP antagonist^17, 43^). Moreover, an increased level of EP3 or TP-independent vasoconstrictor activity of PGE_2_ had been previously found in SHR aortas^43^. However, the present study further suggest a novel mechanism, which involves the up-regulation of EP3, for the increased vasoconstrictor activity or EDCF-like function of PGI_2_ in rat resistance arteries developed under hypertensive conditions^25, 27, 34^. It should be noted that PGI_2_ effectively activating EP3 along with TP to mediate vasoconstrictor activity, an idea we recently put forward based on results obtained in vessels from TP^−/−^ mice^35^, was further verified in the present study by the effect EP3^−/−^ on PGI_2_’s *in vitro* response. Indeed, the idea of PGI_2_ being able to effectively act on EP3 explains why many of clinically used PGI_2_ analogues, such as iloprost, are also EP3 agonists^44^.

One might have also noted that the EP1 antagonist SC19220 (at 100 μM) was previously found to abolish the part of increased vasoconstrictor activity of PGE_2_ resistant to TP antagonism in SHR aortas^43^, arousing a concern that the absence of effect by the antagonist in our present study might be due to an inadequate amount used. However, in SHR aortas the level of EP1 was down-regulated and in fact, such an effect of SC19220 was not considered to result from EP1 antagonism^43^. In addition, SC19220 is known to antagonize EP1 from 1 μM^45, 46^. As such, the absence of effect by 10 μM SC19220 in the present study could be reasonably considered to result from little, if any functional involvement of EP1^46^. Also, in one of prior reports COX-2 inhibition abolished EDCF activity in SHR mesenteric resistance arteries^17^, which contradicts with the results obtained in our present study. Again, one must also note that the COX-2 inhibitor used in the prior report even inhibits EDCF activity in COX-2^−/−^ mice^47^. In contrast, our current results were not only consistent with findings obtained in COX-1^−/−^ or -2^−/−^ mice, but also those in other vessels of WKYs or SHRs^12, 21, 25, 34, 48, 49^.

In summary, in this study our results not only explicitly demonstrate that EP3 is involved in PGI_2_-evoked vasoconstrictor activity, but also suggest that its up-regulation could, in synergy with that of TP and down-regulation of IP account for the increased contractile activity of PGI_2_ in SHR mesenteric arteries.

## Material and Methods

### Chemicals and solution

L-NAME, ACh, PE, the EP3 antagonist L798106, and the EP1 antagonist SC19220 were purchased from Sigma (St Louis, MO, USA). The COX-1 selective inhibitor FR122047, the IP antagonist CAY10441, the TP antagonist SQ29548, TP agonist U46619, PGI_2_ and standard compounds of COX products were bought from Cayman Chemical (Ann Arbor, MI, USA). The selective COX-2 inhibitor rofecoxib was purchased from US Biological (Salem, MA, USA). L-NAME, PE, ACh, and FR122047 were dissolved in distilled water, while PGI_2_ was dissolved in carbonate buffer (50 mM, pH 10.0). SQ29548, L798106, SC19220, CAY10441, and rofecoxib were dissolved in DMSO at 2,000-fold working concentration (the final concentration of DMSO was 0.05/100, v/v). The concentration of an inhibitor or antagonist used was based on previous reports, in which a selective inhibition of the effect of its intended target was considered to be achieved^23, 35, 45, 46, 50, 51^.

The composition of physiological salt solution (PSS; pH 7.4 with 95%O_2_-5% CO_2_) was as follows (in mM): NaCl 123, KCl 4.7, NaHCO_3_ 15.5, KH_2_PO_4_ 1.2, MgCl_2_ 1.2, CaCl_2_ 1.25, and D-glucose 11.5. The 60 mM K^+^-PSS (K^+^) was prepared by replacing an equal molar of NaCl with KCl.

### Animals and tissue preparation

All procedures were in conformance with the Guide for the Care and Use of Laboratory Animals published by the US National Institutes of Health (NIH Publication No. 85–23, revised 1996), and approved by The Institutional Animal Research and Use Committee of Shantou University.

Male WKYs or SHRs (10–14 wk) were purchased from Vital River (Beijing, China). Prior to each experiment, systemic blood pressure (BP) was measured in each rat, using a computerized noninvasive BP system (Kent Scientific Corporation, Torrington, CT, USA). WKYs with BP < 120/90 mmHg or SHRs with systolic BP > 170 mmHg were included in this study.

The breeder male and female EP3^−/−^ mice (on a C57BL/6 background) were custom produced by View Solid Biotech (Beijing, China), using a CRISPR-Cas9 method that targeted introns flanking exon 1 of the EP3 locus and resulted in deletion of a DNA fragment of 1,146 bp, which contains the whole exon 1 (coding for the 1–276^th^ of 362 amino acids). C57BL/6 wild-type (WT) mice were purchased from Vital River (Beijing, China). The deletion of EP3 in each EP3^−/−^ mouse was further confirmed by genotyping with tail biopsy. PCR primers were 5′-TCC CAG ATG TGA GTA TCA TAT G-3′ (sense), and 5′-TAG CTA CCT GAG AAC CTT TAG TG-3′ (anti-sense). The expected PCR product sizes are 1,749 bp in WT, while 603 in EP3^−/−^ mice. Male WT and EP3^−/−^ mice (8–12 wk) were used for experimental purpose.

Rats or mice were killed by CO_2_ inhalation. Rat mesenteric branches or mouse abdominal aortas were isolated and dissected free of adherent tissues with the help of a binocular microscope.

### Analyses of vasomotor reaction

For analyses of vascular function, 2^nd^ generation branches of rat mesenteric arteries or mouse abdominal aortas were cut into 1 mm rings. Vasomotor reaction was measured as described previously^34^. Briefly, the vascular ring was mounted between two tungsten wires in an organ bath filled with PSS aerated with 95%O_2_-5% CO_2_ and maintained at 37 °C. One wire was stationary, whereas the other was connected to an AE801 force transducer (Kronex, Oakland, USA). For some experiments, the endothelium of rat vessels was denuded as previously described^35^. Thereafter, vessels were stimulated with 60 mM K^+^ every 15 minutes, and the resting tension was adjusted stepwise to an optimal level (~250 mg for rat mesenteric resistance arteries, while ~300 mg for mouse abdominal aortas), at which point the response to 60 mM K^+^ was maximal and reproducible.

To remove the influence of NO, vessels were treated with the NOS inhibitor L-NAME (1 mM), under which the response of arteries appears similar to that of eNOS^−/−^ mice^19^. Inhibitors or solvents were added 30 min before the vessel was contracted with an agonist and was kept in the solution throughout the experiment. The response elicited by an agonist under the baseline condition was expressed relative to that of 60 mM K^+^, while that during the contraction evoked by PE (3 μM in endothelium-denuded or L-MAME-treated rat vessels to achieve a sustained contraction of 90–110% that by 60 mM K^+^, or at concentrations otherwise indicated) was expressed relative to the value immediately prior to the application of the agent.

### Assay of 6-keto-PGF_1α_

The PGI_2_ metabolite 6-keto-PGF_1α_ was measured with an EIA kit^12^. Briefly, after rinsed of blood components, mesenteric arterial branches were incubated with PSS at 37 °C for 30 min, followed by exposure to PSS (100 μl) or ACh (10 μM) in 100 μl PSS (37 °C) for 15 min. Thereafter, vessels were taken out, and the reaction solutions were diluted with PSS (1:50), and 100 μl of final solution was used for 6-keto-PGF_1α_ measurement, according to instructions of the manufacturer. The amount of 6-keto-PGF_1α_ was expressed in ng per mg of wet tissue.

In addition, the production of 6-keto-PGF_1α_ in SHR mesenteric branches was verified with HPLC-mass spectroscopy (HPLC-MS), by which signals of other COX-derived products could be simultaneously monitored^40^. Briefly, after being incubated with PSS at 37 °C for 30 min, SHR mesenteric branches (all branches of a single rat were pooled for one measurement) were additionally treated in 1,000 μl PSS (37 °C) or that containing ACh (10 μM) for 15 min. The extraction of COX-derived products and its HPLC-MS detection were performed as described previously^52^.

### Real-time PCR

The expression of EP3 was detected with real-time PCR. The preparation of total RNA from rat mesenteric arteries and RT reactions were performed as described elsewhere previously^12^. First-strand cDNA was synthesized using total RNA (250 ng) and oligo(dT)15 primers (TaKaRa; Dalian, China).

The primers for EP3 were: 5′-TCG CCG CTA TTG ATA ATG ATG C-3′ (sense), 5′-GCA CTC CTT CTC CTT TCC CAT CT-3′ (antisense). Those for β-actin (internal control) were 5′-CCG TAA AGA CCT CTA TGC CAA CA-3′ (sense) and 5′-CGG ACT CAT CGT ACT CCT GCT-3′ (antisense).

### Western blot

Expressions of TP, IP, and β-actin (internal controls) were detected by Western blot. Anti-TP (polyclonal; rabbit; 1:3,000), and anti-IP (polyclonal; rabbit; 1:2,000) antibodies (polyclonal; rabbit; 1:3,000) were purchased from Cayman Chemical, while the anti-β-actin antibody (polyclonal; rabbit; 1:2,000) was bought from Santa Cruz Biotechnology (Santa Cruz, CA, USA). Immunocomplexes were visualized with reaction solution from an ECL Prime detection kit (GE Healthcare, Shanghai, China), and detected using Kodak X-ray film (XBT-1; Xiamen, China).

### Data Analysis

Data were expressed as means ± SEM from n numbers or pools of vessels from different animals. Student’s t-test was used to compare the difference between two means for statistical evaluation. When more than two means were compared, one-way or two-way ANOVA followed by Bonferroni’s post-hoc test was used. P < 0.05 was considered to be statistically significant.

## Electronic supplementary material

Supplemental information
