# Supplementary material for: Increased role of E prostanoid receptor-3 in prostacyclin-evoked contractile activity of spontaneously hypertensive rat mesenteric resistance arteries
Source: Sci Rep. 2017 Aug 21;7:8927. doi: 10.1038/s41598-017-09288-w (PMC5566542; doi:10.1038/s41598-017-09288-w)

Increased role of E prostanoid receptor-3 in prostacyclin-evoked contractile activity of spontaneously hypertensive rat mesenteric resistance arteries

Bin Liu[1], Mengyi Zhan[1], Yingzhan Zhang[1], Hui Li[2], Xiangzhong Wu[1], Fengfeng Zhuang[3], Wenhong Luo[2] and Yingbi Zhou* [1]

1. Cardiovascular Research Center, Shantou University Medical College, Shantou, China; 2. The Central Lab, Shantou University Medical College, Shantou, China; 3. Beijing View Solid Biotechnology, Beijing, China

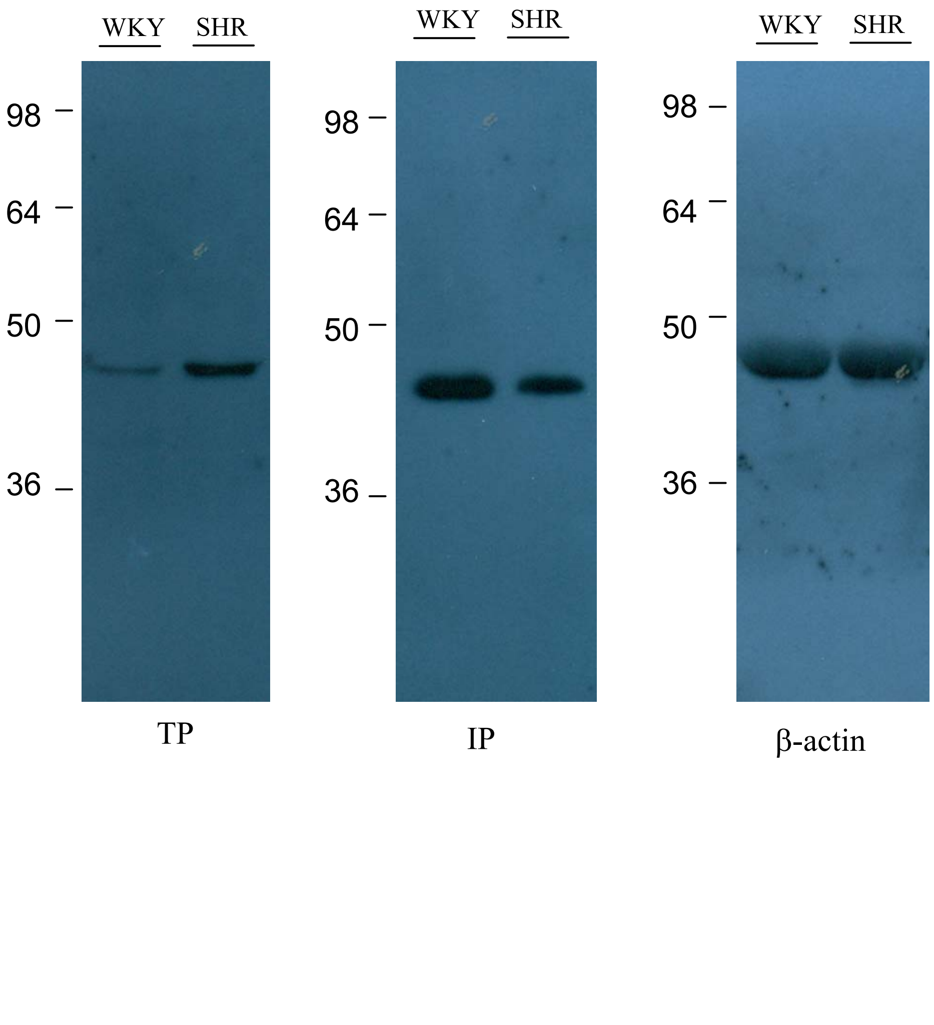

Supplement: Supplementary file 1 — Supplemental information [file 41598_2017_9288_MOESM1_ESM.pdf]
